# Atomic Force Microscopy Study of the Effect of an Electric Field, Applied to a Pyramidal Structure, on Enzyme Biomolecules

**DOI:** 10.3390/jfb13040234

**Published:** 2022-11-10

**Authors:** Yuri D. Ivanov, Vadim Y. Tatur, Ivan D. Shumov, Andrey F. Kozlov, Anastasia A. Valueva, Irina A. Ivanova, Maria O. Ershova, Nina D. Ivanova, Igor N. Stepanov, Andrei A. Lukyanitsa, Vadim S. Ziborov

**Affiliations:** 1 Institute of Biomedical Chemistry, 119121 Moscow, Russia; 2 Joint Institute for High Temperatures of the Russian Academy of Sciences, 125412 Moscow, Russia; 3 Foundation of Perspective Technologies and Novations, 115682 Moscow, Russia; 4 Moscow State Academy of Veterinary Medicine and Biotechnology Named after Skryabin, 109472 Moscow, Russia; 5 Faculty of Computational Mathematics and Cybernetics, Moscow State University, 119991 Moscow, Russia

**Keywords:** atomic force microscopy, horseradish peroxidase, protein aggregation, applied electric field

## Abstract

The influence of an external constant strong electric field, formed using a pyramidal structure under a high electric potential, on an enzyme located near its apex, is studied. Horseradish peroxidase (HRP) is used as a model. In our experiments, a 27 kV direct current (DC) voltage was applied to two electrodes with a conducting pyramidal structure attached to one of them. The enzyme particles were visualized by atomic force microscopy (AFM) after the adsorption of the enzyme from its 0.1 µM solution onto mica AFM substrates. It is demonstrated that after the 40 min exposure to the electric field, the enzyme forms extended structures on mica, while in control experiments compact HRP particles are observed. After the exposure to the electric field, the majority of mica-adsorbed HRP particles had a height of 1.2 nm (as opposed to 1.0 nm in the case of control experiments), and the contribution of higher (>2.0 nm) particles was also considerable. This indicates the formation of high-order HRP aggregates under the influence of an applied electric field. At that, the enzymatic activity of HRP against its substrate 2,2′-azino-bis(3-ethylbenzothiazoline-6-sulfonate) (ABTS) remains unaffected. These results are important for studying macroscopic effects of strong electromagnetic fields on enzymes, as well as for the development of cellular structure models.

## 1. Introduction

Electric fields play a crucial role in protein-protein and protein-lipid interactions (membrane processes), and in cell functioning. A large number of studies on the influence of electromagnetic [1,2,3,4,5] and magnetic [6,7,8] fields (both constant and alternating) on enzymes was reported, and in many of these works, horseradish peroxidase (HRP) is considered since it is a thoroughly studied model object [2,3,6,7,8,9,10,11,12]. For this enzyme, the effects of strong constant and alternating electromagnetic fields were studied. Zhang et al. [13] found that strong (30 kV/cm) pulsed electric fields have an inactivation effect on HRP, changing its conformation. Constant electric fields also affect HRP significantly. Among such papers, the study by Yang et al. on the influence of high-strength constant electric fields on the horseradish peroxidase activity [14] should be noted. These authors demonstrated an increase in the enzymatic activity of HRP at an electric field strength (*E*) in the range 2 × 10^5^ < *E* ≤ 6 × 10^5^ kV/cm, while no experiments were performed for stronger electric fields.

Two important points should be noted. Firstly, in a biological cell membrane, the electric field strength is about 2 × 10^6^ V/m. Secondly, these fields can be localized: for instance, as in protein pores of a funnel-shaped structure [15]. Thus, the influence of electric fields generated near model funnel-shaped structures, e.g., pyramidal or conical, on proteins and enzymes represents an actual task of modern biomedicine. Herein, a setup based on a pyramidal electrode has been used, and a solution of HRP enzyme was incubated near the apex of the pyramid. The strength of the electric field, generated with this element, was ~2 × 10^6^ to 10^7^ V/m, being similar to the one generated by a cell membrane. In addition, it should be mentioned that pyramidal structures can be used in biosensors [16].

Atomic force microscopy (AFM) was used as a research method since it allows visualization of single biological macromolecules [17,18]. AFM is a high-resolution technique, which is very useful in studying single polymer macromolecules [19]—in particular, proteins [18,20,21,22,23] and nucleic acids [24,25]—at a nanoscale. The height resolution of AFM is very high (~0.1 nm) and enables obtaining images of single biological macromolecules and their aggregates [9,10,11,12,21]. AFM was employed to study the influence of very weak electromagnetic fields on the aggregation state of HRP [9].

In the present work, HRP was used as a model enzyme, since it is characterized in much detail in the literature and has been used in studying the effect of electromagnetic fields on proteins [2,3,4,5,9,10,11,12,26]. HRP is a heme-containing enzyme glycoprotein with a molecular weight of 40–44 kDa [27,28], containing 18–27% structure-stabilizing carbohydrate residues [28,29]. In micromolar aqueous solutions, HRP tends to form aggregates [30]. At a concentration of ~10^−7^ M in a buffer solution, HRP exists as a mixture of monomers and aggregates [9].

Herein, by AFM, we have for the first time demonstrated that a 40 min exposure of 0.1 µM solution of HRP to a strong (27 kV) constant electric field applied to a pyramidal structure causes an increase in the enzyme aggregation on mica, which occurs in the form of extended structures. At the same time, its enzymatic activity remains unaffected. The results obtained are of great importance for the studying of enzyme and cellular structures, as well as for better understanding of the effect of strong electric fields on other enzyme systems.

## 2. Materials and Methods

### 2.1. Chemicals and Enzyme

Peroxidase from horseradish and its substrate 2,2′-azino-bis(3-ethylbenzothiazoline-6-sulfonate) (ABTS) were from Sigma (St. Louis, MO, USA; enzyme Cat.#: P6782; ABTS Cat.#: A1888). Disodium hydrogen orthophosphate (Na_2_HPO_4_), citric acid and hydrogen peroxide (H_2_O_2_) were of analytical or higher grade (Reakhim, Moscow, Russia). All solutions were prepared using deionized ultrapure water (of 18.2 MΩ × cm resistivity) obtained with a Simplicity UV system (Millipore, Molsheim, France).

### 2.2. Experimental Setup

**Figure 1:**
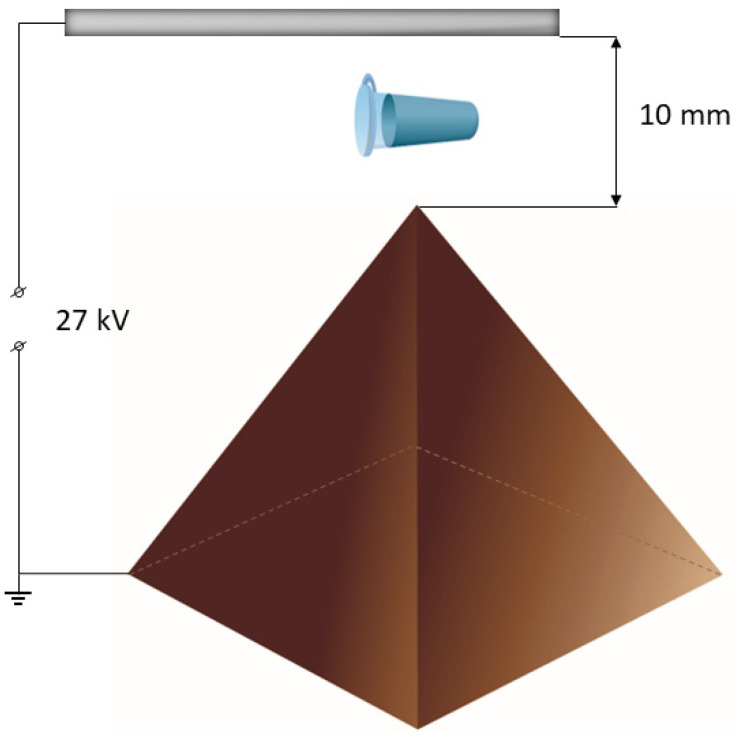
Experimental setup. The distance between the apex of the pyramid (brown) and the electrode (grey) is 10 mm. The test tube (turquoise) with the enzyme sample (0.1 µM HRP solution) was placed over the apex of the pyramid. The pyramid was under a potential of 27 kV relative to the ground.

The experimental setup used in the present study is schematically shown in Figure 1.

The pyramidal structure had a height of 3.5 cm, a base edge size of 4.5 cm, and an apex angle of 52°. A 27 kV DC voltage was applied to the electrodes, and the pyramid was attached to one of them. The pyramid material was copper-coated textolite. The distance between the apex and the electrode was 1 cm. A standard 1.7-mL Eppendorf-type polypropylene test tube, containing 1 mL of 0.1 µM (10^−7^ M) aqueous HRP test solution in 2 mM Dulbecco’s modified phosphate buffered saline, is placed over the apex of the pyramid and incubated for 40 min. The control sample is incubated 2 m away from the pyramidal structure.

### 2.3. AFM Sample Preparation

Samples for AFM scanning were prepared by direct adsorption onto the surface [31]. Freshly cleaved mica plates (SPI, West Chester, PA, USA) were used as AFM chips. During sample preparation, each AFM chip was immersed in Eppendorf-type polypropylene tubes containing 800 µL of a 0.1 µM HRP aqueous solution. Then the AFM chips were incubated in the studied solutions for 10 min at room temperature using a shaker (600 rpm). After incubation, each AFM chip was washed in 1 mL of ultrapure water, followed by drying in the air.

### 2.4. AFM Measurements

Samples—mica substrates with adsorbed HRP molecules from control and working solutions—were scanned in a semi-contact mode in the air at a temperature of 25 °C with a Titanium atomic force microscope (NT-MDT, Zelenograd, Russia; the microscope pertains to the equipment of the “Human Proteome” Core Facility of the Institute of Biomedical Chemistry, supported by the Ministry of Education and Science of Russian Federation, Agreement 14.621.21.0017, unique project ID: RFMEFI62117X0017). The microscope was equipped with NSG10 cantilevers (“TipsNano”, Zelenograd, Russia; tip radius 10 nm; resonant frequency 140–390 kHz, force constant 3.1–37.6 N/m; cantilever length, 125 ± 10 µm; cantilever width, 27 ± 7.5 µm; cantilever thickness, 2.75 ± 1 µm). The scanning rate was 1 Hz. The scanning area was 1 × 1 μm^2^ or 2 × 2 μm^2^ (256 × 256 points), and at least 10 scans were made for each sample in different parts of the mica surface. The microscope was calibrated in height using an aTGZ1 calibration grating (NT-MDT, Moscow, Zelenograd, Russia, step height 21.4 ± 1.5 nm).

The total number of visualized objects for each sample was at least 200. The distribution of the relative number of objects with height *ρ*(*h*) was calculated as described by Pleshakova et al. [32]. AFM scanning and further treatment of AFM images (subtraction of 2nd order surface) were performed using the standard NOVA Px software (NT-MDT, Moscow, Zelenograd, Russia) supplied with the microscope. The number of objects on the AFM chip surface was calculated, and the relative distribution of objects by heights was obtained using software developed in the Institute of Biomedical Chemistry together with the Foundation of Perspective Technologies and Novations.

### 2.5. Spectrophotometry Measurements

HRP activity monitoring was estimated based on the approach detailed by Sanders et al. [33] employing ABTS as a substrate. ABTS assay should be performed at pH 5.0 [34]. The rate of change in solution absorbance at 405 nm was measured with an Agilent 8453 UV-visible spectrophotometer (Agilent Technologies Deutschland GmbH, Waldbronn, Germany) as described in our previous studies [9,10,11,12,26].

## 3. Results

### 3.1. Atomic Force Microscopy

**Figure 2:**
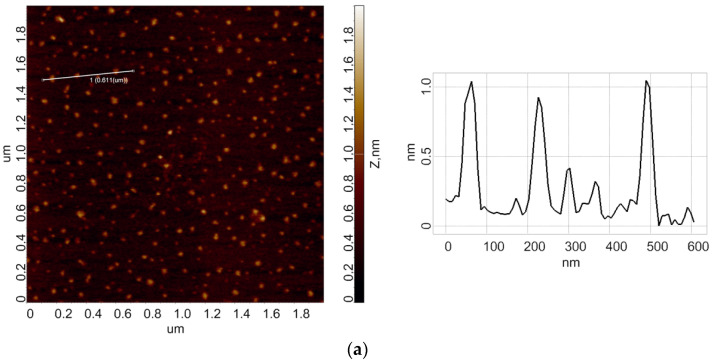

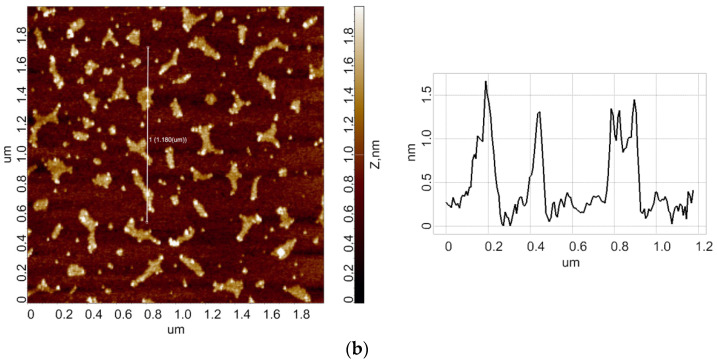
Results of AFM experiments. Typical AFM-images of the surface of mica substrates with adsorbed HRP macromolecules (left) and cross-section profiles (right), corresponding to the lines in the AFM images. The analyzed HRP solution was incubated either 2 m away from the pyramid (**a**); control solution) or above the pyramid (**b**). The pyramid was under a potential of 27 kV relative to the ground.

In the working experiments, the HRP solution was incubated for 40 min over the apex of the pyramid, to which a high (27 kV) voltage was applied. In the control experiments, the enzyme sample was placed 2 m away from the pyramid. Below, the results of AFM scanning of bare mica substrates, obtained after their incubation in the analyzed HRP solutions, are presented. Figure 2 displays typical AFM images of mica-adsorbed HRP, obtained in the working (when the samples were placed over the pyramid apex) and in control experiments.

As can be seen from Figure 2a, in the control experiments, compact isolated objects were observed on the mica substrate surface. In the working experiments, when the enzyme solution was incubated over the apex of the pyramid under high voltage (Figure 2b), extended objects of various shapes were observed. 

**Figure 3:**
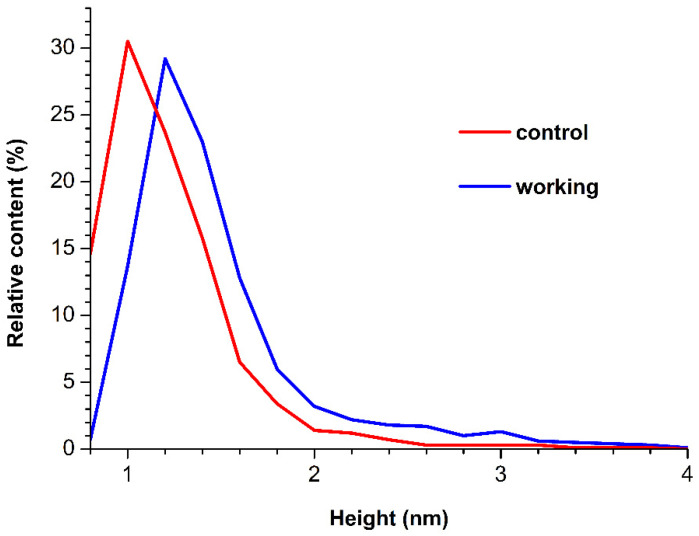
Relative distributions of the mica-adsorbed HRP particles with height *ρ*(*h*) were obtained for the HRP samples incubated either 2 m away from the pyramid (red; control experiment), or above the pyramid (blue, working experiment). The pyramid was under a potential of 27 kV relative to the ground.

The corresponding density functions *ρ*(*h*) for the working and control experiments were calculated and plotted. Figure 3 displays the so-obtained *ρ*(*h*) curves.

The *ρ*(*h*) curves shown in Figure 3 indicate that, for the control sample, the distribution reaches its global maximum at a height *h* = 1.0 nm. In the working experiments with the sample incubated over the apex of the pyramid under high voltage, the height distribution maximum is 1.2 nm. As was justified previously, the 1.0-nm-high HRP particles correspond to the monomeric form of the enzyme [9,11,26]. Accordingly, particles with substantially greater heights (*h* > 1.6–1.8 nm) correspond to aggregated HRP on mica. Moreover, in comparison with the control experiment, an increased contribution of objects with heights *h* > 1.8 nm to the right wing of the distribution is observed for the sample exposed to the 27 kV field. This indicates an increase in the aggregation of the enzyme after its 40 min exposure to the 27 kV electric field.

### 3.2. Spectrophotometry

**Figure 4:**
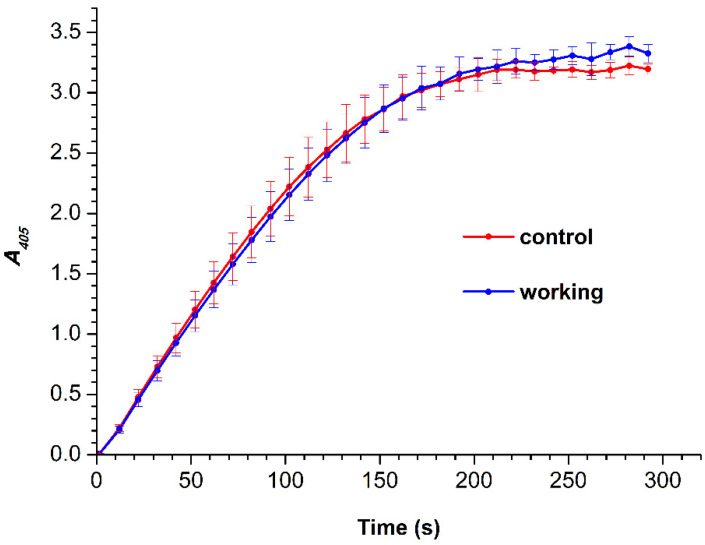
*A_405_*(*t*) curves were obtained for the HRP samples incubated either 2 m away from the pyramid (red; control experiment), or above the pyramid (blue, working experiment). The pyramid was under a potential of 27 kV relative to the ground.

Figure 4 displays time dependencies of absorbance of solutions containing HRP, incubated in the experimental setup, and its substrate ABTS at 405 nm (*A_405_*(*t*) curves).

One can see that in the 0–220 s time interval, the *A_405_*(*t*) curve recorded in the working experiment (blue) is barely distinguishable from that recorded in the control experiment (blue). After 220 s, both curves reach the plateau. At that, the *A_405_* values recorded for the working experiment are slightly higher than those recorded in the control experiment, but this difference does not exceed the values of the experimental error. Accordingly, one can state that no change in the activity of HRP against ABTS after its 40 min exposure to a 27 kV electric field has been revealed during a 300 s observation.

## 4. Discussion

In the present work, we have studied the influence of a pyramidal structure, to which an external potential was applied (hence, a high-intensity electric field was formed), on the properties of the HRP enzyme. The strength of such a field was close to that observed in a cell membrane (2 × 10^6^ V/m).

Moreover, it was shown that the enzymatic activity of the HRP increases under strong electric fields [14]. This may take place owing to a change in the enzyme structure. In the present study, the influence of a strong electric field generated with the use of using a pyramid was studied. Exposition of the HRP sample to such a field has led to a significant change in protein aggregation on mica, which manifested itself by forming extended structures. At the same time, its enzymatic activity remained unaffected. These extended structures can appear when several enzyme globules stick together. This phenomenon has not been observed in the control experiments. It can be explained by a complex effect of changes in the hydration of the enzyme macromolecule, and these changes can affect the enzyme structure [35,36], thus influencing the interactions of individual enzyme macromolecules with the substrate, with each other (on the substrate surface), and with the solvent [37]. Furthermore, such a change in the hydration shell can also be caused by an increasing conversion of ortho/para water isomers in a strong electric field owing to the Stark effect [38]. This increased aggregation can also be explained by the fact that under the above-mentioned conditions, the structure of the enzyme (and its hydration) influences the interaction energy determined by the protein-substrate and protein-protein interaction so that it becomes greater than the interaction energy of a simple protein globule with the substrate. This energy gain determines the organization of extended protein structures observed in our experiments. Taking into account that no change in the enzymatic activity of HRP has been observed, one can conclude that the enzyme’s active site is unaffected by the 27 kV electric field after 40 min exposure.

## 5. Conclusions

The present research on the influence of the electric field will contribute to further studies on the interactions between enzyme macromolecules and on cell functioning. Our results should also be considered upon studying the influence of strong electromagnetic fields on the functioning of membrane systems and cells. In addition, the results of our study are useful for estimating the impact of strong electromagnetic fields on other enzyme systems. It should be noted that a number of devices, such as biosensors, can include high-voltage circuits, which can influence enzymes and other biological macromolecules. In this respect, our results emphasize the importance of further research in this direction. Accordingly, our findings can be considered an improvement to the interpretation of results of analysis of enzymatic systems in novel biosensors with high-voltage circuits. This is essential for rapid diagnosis of cardiovascular, oncological, and other pathologies in humans with high sensitivity.

## Data Availability

Correspondence and requests for materials should be addressed to Y.D.I.

## Abbreviations

ABTS: 2:2′-azino-bis(3-ethylbenzothiazoline-6-sulfonate); AFM, atomic force microscopy; DC, direct current; HRP, horseradish peroxidase.
